# Experimental Demonstration of Printed Graphene Nano-flakes Enabled Flexible and Conformable Wideband Radar Absorbers

**DOI:** 10.1038/srep38197

**Published:** 2016-12-07

**Authors:** Xianjun Huang, Kewen Pan, Zhirun Hu

**Affiliations:** 1 School of Electrical and Electronic Engineering, University of Manchester, Manchester, M13 9PL, UK

## Abstract

In this work, we have designed, fabricated and experimentally characterized a printed graphene nano-flakes enabled flexible and conformable wideband radar absorber. The absorber covers both X (8–12 GHz) and Ku (12–18 GHz) bands and is printed on flexible substrate using graphene nano-flakes conductive ink through stencil printing method. The measured results show that an effective absorption (above 90%) bandwidth spans from 10.4 GHz to 19.7 GHz, namely a 62% fraction bandwidth, with only 2 mm thickness. The flexibility of the printed graphene nano-flakes enables the absorber conformably bending and attaching to a metal cylinder. The radar cross section (RCS) of the cylinder with and without absorber attachment has been compared and excellent absorption has been obtained. Only 3.6% bandwidth reduction has been observed comparing to that of un-bended absorber. This work has demonstrated unambiguously that printed graphene can provide flexible and conformable wideband radar absorption, which extends the graphene’s application to practical RCS reductions.

Radar absorbers are widely used in both civil and military applications such as EMI, EMC, antenna pattern shaping, stealth technology and etc.^1^,^2^,^3^. Premium absorbers are normally required to have high absorption, wide bandwidth, light weight, low profile and mechanical flexiblility^3^. Traditionally, Salisbury screen and its alike have been widely used for radar absorption. However narrow operating bandwidth or relatively thick substrate due to quarter wavelength requirement has hindered their applications^3^,^4^. Recently, metamaterial inspired and resistive frequency selective surface (FSS) absorbers have been proposed. Metamaterial inspired absorber was firstly proposed in 2008. It was characterized with low profile but it has narrow bandwidth^5^. To increase the bandwidth, multi-resonances were introduced^6^,^7^,^8^,^9^,^10^. However, the bandwidth needs to be further enhanced for practical applications. On the other hand, advantages of combining metamaterial and resistive FSS to obtain wideband and low-profile absorbers were theoretically demonstrated^11^,^12^. The metamaterial resistive FSS absorber that has 0.11λ thickness at lowest frequency and achieved 80% fractional bandwidth from 8 to 19 GHz has been reported^12^. Metamaterial structures combining with lump resistors to achieve enhanced absorption bandwidth were also demonstrated^13^,^14^,^15^. For all of these works, the absorbers typically consist of metal patterns photolithographed on PCB board, which means no flexibility is provided.

With the development of the printed electronics, many conductive inks are developed and can be used to replace conventional conductors^16^,^17^,^18^,^19^,^20^,^21^,^22^. Printable conductive inks have incomparable advantages over conventional metal sheet in radar absorber applications, considering its lightweight, flexibility and mass-production compatibility^16^. With the development of printed electronics, many options such as silver/copper nanoparticles/nanowires^17^,^18^,^19^, conductive polymers^20^,^21^, carbon black and carbon nanotubes^16^,^22^, are possible candidates for constructing printed radar absorbers. Generally highly conductive inks are desirable for printed radar absorbers because non-conductive additives can be added to improve other printing properties such as adhesion, waterproof, flexibility and etc.^16^. Silver nanoparticle inks are advantageous in conductivity^17^, and a flexible inkjet-printed metamaterial absorber was fabricated with it^23^. However, high costs of silver nanoparticles and low fabrication efficiency of inkjet printing have prevented their large-scale applications^16^,^23^. Copper nanoparticle inks are much cheaper but very easy to be oxidized, especially during high-temperature annealing^19^, which considerably limits its applications. Conductive polymers are less conductive and its applications are hindered by their chemical and thermal instability^20^,^21^. Carbon black and carbon nanotubes are cost effective and not easy to be oxidized. Their typical sheet resistance is above 50 *Ω*/sq^16^,^22^, which is slightly higher than desirable value for a radar absorber. Graphene, the first isolated two dimensional carbon material, has many superior properties such as extraordinary high electron mobility, conductivity and thermal conductivity, to name a few^24^. Enabled by graphene high conductivity and electrical tunability, tunable microwave and THz absorbers have been developed based on CVD graphene^25^,^26^,^27^,^28^.

However CVD/exfoliated graphene absorbers need clean room process and are too expensive for practically deployable radar applications. Graphene nano-flakes ink and its printed products are obviously preferable alternative due to its compatibility with mass-production printing process and low fabrication costs^29^,^30^,^31^,^32^,^33^,^34^. In our previous works, we have demonstrated graphene’s advantages in printing highly conductive antennas^31^,^32^, microwave passive components, such as coplanar waveguide (CPW) and open/short circuited resonators, and EM shielding^32^,^33^,^34^, where high conductivity and flexibility of printed graphene were fully utilized. In this work, a graphene ink was purposely developed for radar signal absorption and graphene FSS patterns were printed on flexible substrate to demonstrate that printed graphene can provide desired absorption and wide operational bandwidth for flexible and conformable radar absorption applications.

## Results

**Figure 1:**
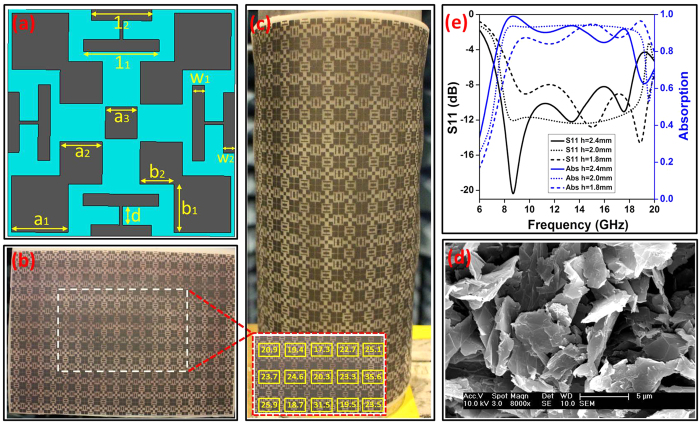
Printed graphene nano-flakes radar absorber. (**a**) Unit cell structure of the absorber and its structural parameters; (**b**) Fabricated graphene absorber; (**c**) Flexible absorber conformably bended and attached to a metal cylinder; (**d**) SEM of top-viewed printed graphene; (**e**) Simulated S_11_ and absorption of graphene absorber under different silicone thickness.

A wideband radar absorber based on modified second-order Saltire cross structure and four H shape coupling additives have been designed, as shown in Fig. 1(a). The absorber consists of printed graphene nano-flakes conductive pattern (dark part in Fig. 1(a)) for impedance matching, flexible silicone substrate (blue part in Fig. 1(a)) and metal ground. The whole absorber was modeled and simulated using commercial software CST MWS^35^. In the simulation, the printed graphene nano-flakes layer was modeled as a resistive sheet with desired sheet resistance 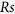. The supporting silicone (Model: Polymax SILONA-Translucent Silicone Sheet 60ShA FDA, thickness 2 mm) has dielectric constant of 2.9 and loss tangent of 0.1, which were measured with Agilent probe (Model: 85070E Dielectric Probe Kit). The absorber was optimized to have as wider absorption band as possible for printed graphene sheet resistance of 20 *Ω*/sq. The geometrical parameters in Fig. 1(a) are: *a*_*1*_ = 3.75 mm, *a*_*2*_ = 2.8 mm, *a*_*3*_ = 2.1 mm, *b*_*1*_ = 3.2 mm, *b*_*2*_ = 2.25 mm, *d* = 1.25 mm, *l*_*1*_ = 5.0 mm, *l*_*2*_ = 4.0 mm, *w*_*1*_ = 0.8 mm, *w*_*2*_ = 0.75 mm. The whole unit cell dimension is 15 mm × 15 mm. Due to the symmetry of the structure, its responses to vertically and horizontally polarized incident waves are the same for planar applications. In this work, the vertical polarization has been chosen for simulations and measurements.

The simulated reflection coefficient (S_11_) and absorption of the absorber with various silicone thicknesses *h*, can be found in Fig. 1(e). It can be seen that the broadest bandwidth can be achieved for *h* = 2 mm. In this case, the optimized absorber has a wideband effective absorption (above 90% absorption) from 8 GHz to 19 GHz, which covers both X and Ku bands. The absolute bandwidth is 11 GHz and fractional bandwidth is 81.5%. It can be found that the absorption band extends to lower frequency with thicker substrate, whereas thinner substrate provides better absorption at higher frequency band. Trade-off can be achieved through optimization. In this work, the thickness of the optimized absorber is less than 0.1λ_o_ at central frequency (13.5 GHz), which is thinner in comparison with similar works^3^,^10^.

To fabricate the printed graphene absorber, both graphene nano-flakes conductive ink and a patterned stencil were developed. The graphene nano-flakes conductive ink preparation and the fabrication of absorber can be found in Method section. The fabricated sample, with size of 300 mm × 200 mm can be found in Fig. 1(b). As both printed graphene and silicone substrate are flexible, the whole absorber is flexible. Such mechanical flexibility is an extremely useful feature for radar absorption applications as it enables conformable stealth. As shown in Fig. 1(c), the absorber was conformably bended and attached to a metal cylinder with radius of 5.9 cm.

A SEM characterization of the printed graphene nano-flakes sample was carried out, as shown in Fig. 1(d). It can be seen that the printed graphene nano-flakes are stacked randomly and the layer is highly porous, similar as our previously screen printed sample before compression^31^,^32^. The sheet resistance can be engineered by compression process to provide desired value to suit the applications^31^,^32^. This is a significant engineering approach as additive functional materials (such as binder, dispersant, surfactant, slip agent, coupling agent and etc.) can be added in the ink for better performances (such as adhesion, viscosity, waterproof, printability and etc.), meanwhile still achieving the desired sheet resistance. Namely, more degrees of freedom on trade-off can be provided by highly conductive graphene nano-flakes compared with other options such as carbon nanotubes, etc. The cross-sectional SEM view of the printed layer is given in Figure S1. The thickness of graphene nano-flakes layer is about t = 11 *μ*m, and sheet resistance of this area is measured to be Rs = 21 *Ω*, so the conductivity of the printed layer is calculated to be σ = 4.33 × 10^3^ S/m.

**Figure 2:**
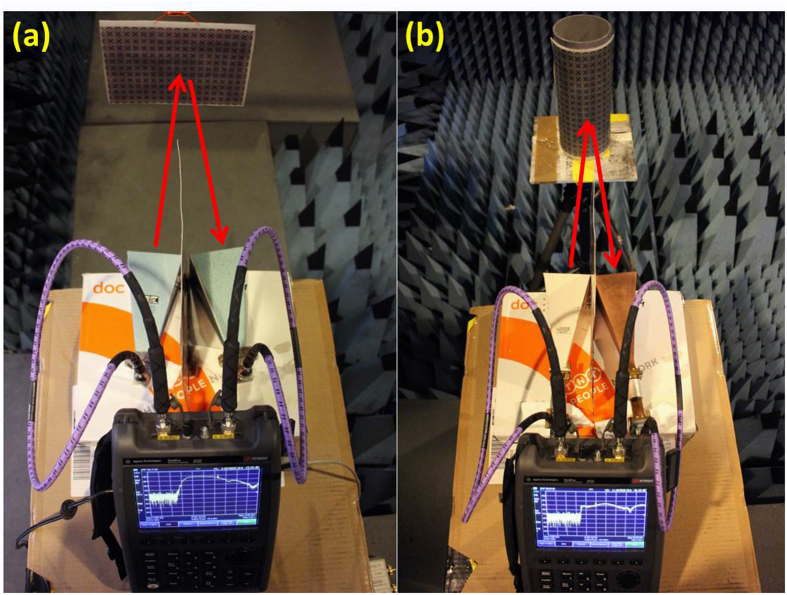
Measurement setup for planar and conformably bended absorbers.

Figure 2 illustrates how the printed graphene absorber was measured for both planar and conformable cases in anechoic chamber. The measurement setup for planar sample is shown in Fig. 2(a). The printed graphene nano-flakes absorber in Fig. 1(b) was attached to a reflective PCB board (Rogers 3015166) for measurement. As it can be seen, a pair of horn antennas working as transmitter/receiver was connected to VNA (Vector Network Analyzer, Fieldfox N9918A, Keysight) to measure the reflection from the sample. An extra PCB board with size of 300 mm × 230 mm was inserted between two antennas for better isolation. The two antennas were slightly angled as seen for better measuring the reflection from the sample. The sample was placed vertically and 0.4 m away from the antenna front faces. As the horn antennas here have limited bandwidth, two pairs of horn antennas were used, one pair for lower band (antenna pair: Standard Gain Horns QSH18, Q-par Angus Ltd) and the other for higher band (antenna pair: Stock No. 5985-99-914-6933, Marconi instruments Ltd) measurements. As the absorber is flexible, a conformably bended case was also measured in anechoic chamber, shown in Fig. 2(b). Similar setup as in Fig. 2(a) was made and the absorber was bended and attached to a metal cylinder with radius of 5.9 cm. The distance of the cylinder (front point) to antenna front faces is still 0.4 m. The same two bands were measured for the conformable absorber.

**Figure 3:**
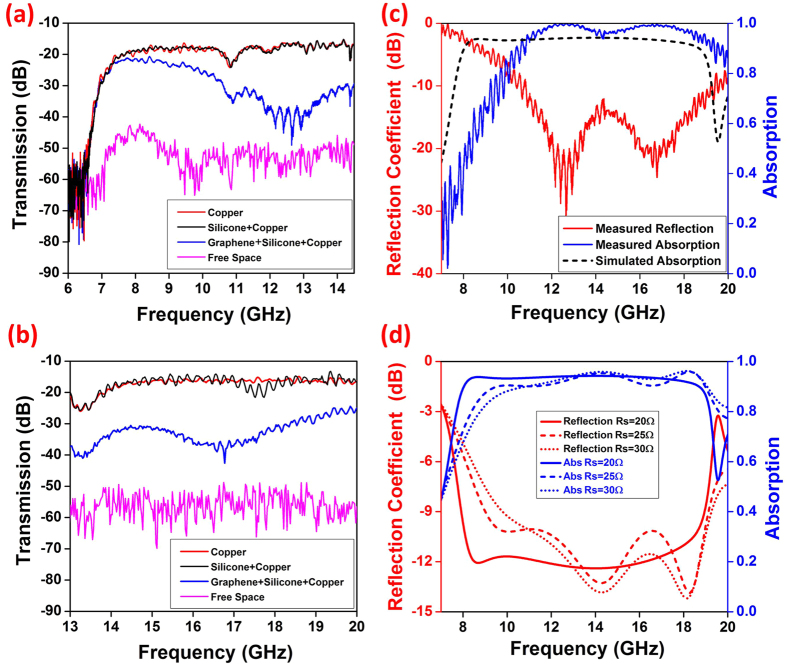
Measured and simulated results for planar printed graphene absorber. (**a**) Transmission coefficient comparison of different samples in lower band; (**b**) Transmission coefficient comparison of different samples in higher band; (**c**) Measured reflection coefficient and absorption of graphene absorber in the combined band and comparison with simulated one; (**d**) Reflection coefficient and absorption of simulated absorber with various sheet resistance.

The measurements were conducted from 6 GHz to 14 GHz (lower band) and from 13 GHz to 20 GHz (higher band) to experimentally examine the RCS of the printed graphene wideband absorbers. In Fig. 3, the measured RCS of the printed graphene nano-flakes absorber in both bands and combined band results are presented. As shown in Fig. 3(a), the lower band reflections for 4 different cases were measured to prove the mechanism of the absorber. Due to the cutoff effect of the waveguide, there is an obvious cut-off in the region of 7–8 GHz. First, the case without any sample present was measured and indicated as free space in Fig. 3(a). It can be seen that the transmission coefficient between the two horn antennas is lower than −50 dB. This transmission is mainly from the direct coupling between the two nearby antennas as well as minor reflection of the anechoic chamber walls. This low level transmission demonstrates that a good isolation was provided by the inserted double-sided metalized PCB board between the two antennas. Next, the copper PCB board and then the same board attached with silicone in front (were measured in the same position (0.4 m away, facing the antennas), respectively. As shown in the Fig. 3(a), these two samples have almost the same transmission coefficients, revealing that the silicone itself cannot act as absorber. Finally, the silicone substrate was replaced by the printed graphene nano-flakes absorber at the same position and measured. By comparing these three cases (copper PCB board, silicone substrate attached on the copper PCB board and printed graphene absorber attached on the copper PCB board), it can be found that the transmission coefficient of the absorber attached on the copper PCB board is much lower than that of other two cases, revealing a satisfactory electromagnetic wave absorption by the absorber. Taking the 12–13 GHz performance as an example, the transmission coefficient from the copper PCB board is around −18 dB to −16 dB, almost the same as that when silicone substrate is attached to the copper PCB board. However, when the silicone is replaced by the absorber, the transmission coefficient reduces significantly to around −40 dB. The absorber provides more than 20 dB attenuation. From the difference of the transmission coefficients, it can be concluded that it is the printed graphene nano-flakes pattern that has absorbed the electromagnetic wave, which leads to significant reduction of RCS.

The same measuring procedure for the planar absorber has been conducted in the higher frequency band from 13 GHz to 20 GHz, and the results are shown in Fig. 3(b). Similar conclusions can be drawn as those from Fig. 3(a), i.e., when the absorber was placed, a significant RCS reduction can be achieved. For a better view on the wideband property of the absorber, these two bands results were combined and shown in Fig. 3(c). In Fig. 3(c), the reflection coefficient of the absorber is obtained from the measured transmission differences between copper PCB board reflector and graphene absorber. The absorption is calculated with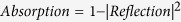. As it can be seen, the effective absorption bandwidth is from 10.4 GHz to 19.7 GHz, indicating an absolute bandwidth of 9.3 GHz and 61.8% fractional bandwidth. The measured results have also been compared with the simulated ones in Fig. 3(c). As it can be seen, the simulation results show better absorption at lower band. This discrepancy is believed to be caused by the sheet resistance differences between the fabricated sample and simulated one. A test on sheet resistance of the printed absorber was conducted to verify whether the fabricated sample has the same sheet resistance as designed (20 *Ω*/sq). As it can be seen in the insertion of Fig. 1(c), the sheet resistance among the central area of the absorber was measured using four point probe system (Jandel Engineering Limited, RM3000). From measurements, it can be seen that the sheet resistance of the absorber is generally close to the expected 20 *Ω*/sq. However, it varies in different sections due to the inconsistency of thickness in the manually sputtered graphene nano-flakes layer. To further examine the cause of this discrepancy, simulations on the influence of sheet resistance variation of graphene nano-flakes layers were carried out. As it can be seen in Fig. 3(d), when the sheet resistance of graphene nano-flakes layer varies from 20 *Ω*/sq to 30 *Ω*/sq, the lower band absorption reduces. As the printed graphene pattern was designed for 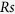 = 20 *Ω*/sq, the larger the differences on sheet resistance, the more deterioration on lower band performance will be. From this analysis, it can be concluded that the differences on simulation and measured results was mainly caused by the non-uniformity of sheet resistance distribution of the printed graphene patterns.

**Figure 4:**
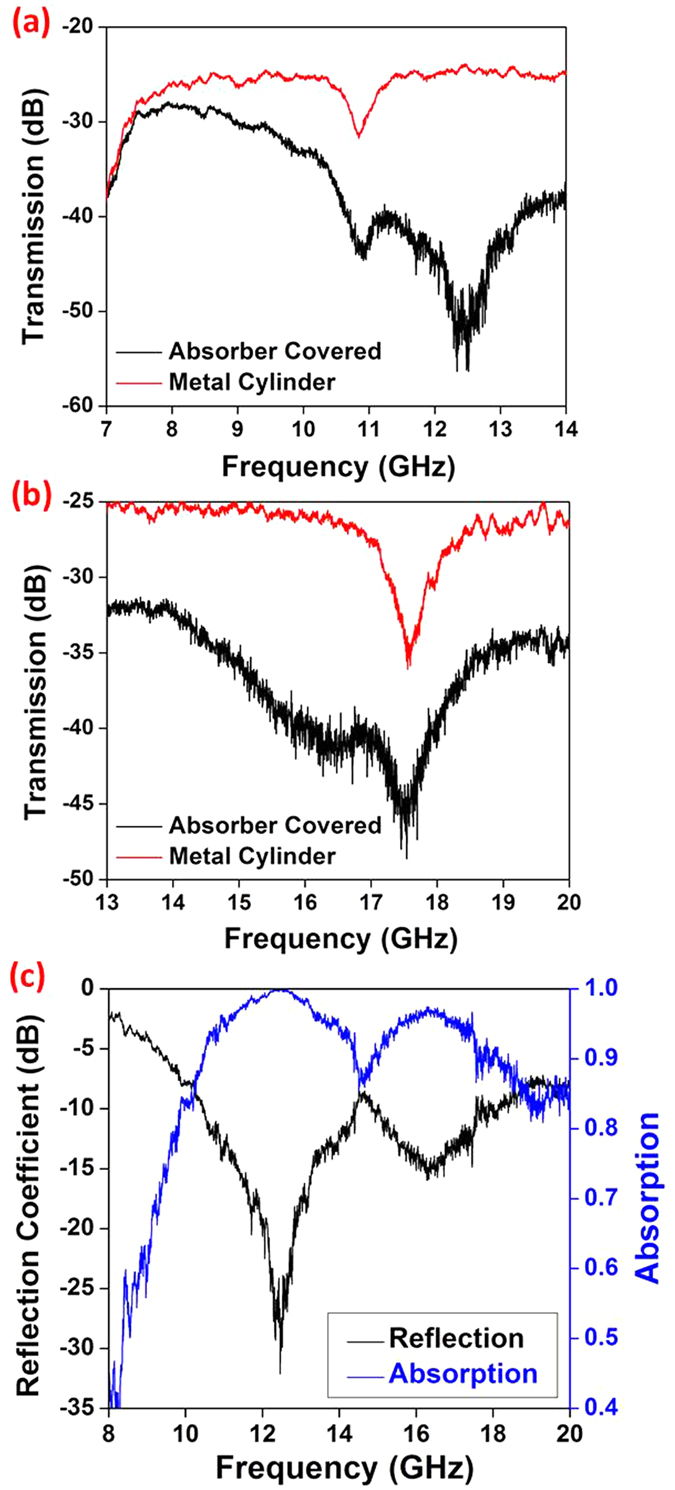
Experimental demonstration of flexibility and conformability of the printed graphene nano-flakes absorber. (**a**) Transmission coefficient comparison of metal cylinder with/without absorber coverage in lower band; (**b**) Transmission coefficient comparison of metal cylinder with/without absorber coverage in higher band; (**c**) Measured reflection coefficient and absorption of bended graphene absorber from 8 GHz to 20 GHz.

For radar absorbers, it would be highly desirable that the absorbers can be mechanically flexible and conformable so to cover differently shaped objects. For this purpose, flexibility and conformability of the printed graphene nano-flakes radar absorber has been investigated. The measurement setup for the conformable case has been explained in Fig. 2(b). The transmission coefficients of the metal cylinder as well as the metal cylinder covered with printed graphene nano-flakes absorber were measured and shown in Fig. 4. The lower band and higher band transmission coefficients with and without absorber can be found in Fig. 4(a) and (b), respectively. It can be seen that the absorber coverage has significantly reduced the RCS of the metal cylinder. For example from 12 to 13 GHz in Fig. 4(a), the transmission coefficient from the metal cylinder is about −25 dB, whereas it reduces to lower than −48 dB, bringing above 23 dB attenuation. Similarly from 16 to 17 GHz in Fig. 4(b), the coverage of the absorber introduces about 15 dB attenuation. The results from the two bands are again combined and shown in Fig. 4(c). It can be seen that the effective absorption bandwidth is from 9.8 GHz to 17 GHz and a fractional bandwidth is 58.2%. Even though the absorption bandwidth reduces slightly by 3.6% compared with the planar case, the wideband absorption is still maintained. From this comparison, it can be concluded that the printed graphene nano-flakes flexible absorber can be conformably adapted to objects and provide effective RCS reduction.

## Discussion

We have applied printed graphene nano-flakes to radar absorbers for RCS reduction in this work. A wideband absorber has been designed, which consists of patterned printed graphene nano-flakes layer, silicone substrate and backed metal. The absorber is fabricated with stencil printing method and the graphene nano-flakes ink are sputtered on silicone layer to form patterns through stencil with the help of air blaster. The absorber has been measured in anechoic chamber and experimentally demonstrated to provide effective absorption from 10.4 GHz to 19.7 GHz and a fractional bandwidth of 61.8%, with thickness only around 2 mm. The printed graphene nano-flakes absorber is mechanically flexible and its performance when bended to cover a metal cylinder have been measured to characterize its conformability. The measurement results show that only 3.6% reduction of the effective absorption bandwidth due to bending. The maintained wideband absorption reveals the potential applications of the printed graphene nano-flakes radar absorber in providing RCS reduction for objects of various shapes. In conclusion, this work has demonstrated unambiguously that printed graphene nano-flakes ink can be used not only for wideband radar absorption, but also flexibility and conformability.

## Methods

### Preparation of graphene nano-flakes ink

**Figure 5:**
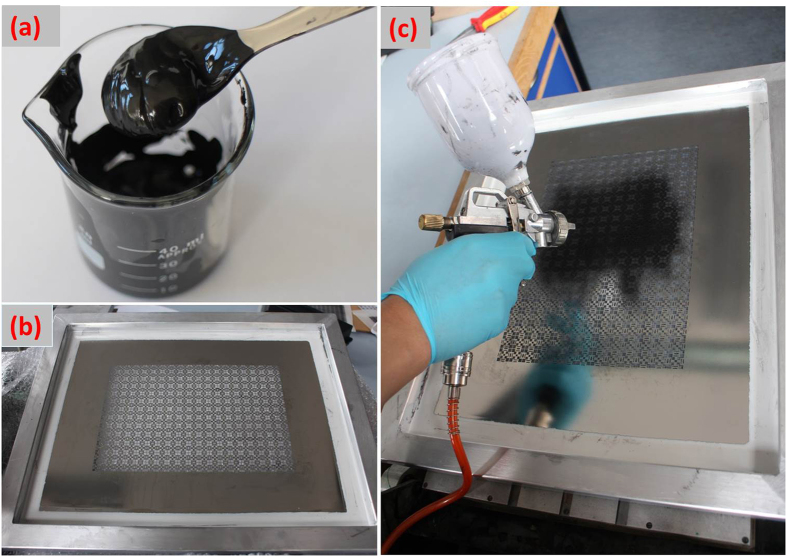
Prepared graphene nano-flakes ink and absorber fabrication. (**a**) Graphene nano-flakes ink; (**b**) Stencil fabricated with laser cutting technology; (**c)** Absorber fabrication with air blaster to sputter ink through stencil.

3 g expanded graphite (EG) was added into a mixture solution of 15 ml ethylene glycol and 45 ml ethanol. Non-ionic polymer-type surfactant was added to improve the dispersion of graphene nano-flakes. After stirring for 20 min, the dispersion was treated with sonication at 820 W with a cooling system for 24 hours. After that, the dispersion was heated on a hot plate with continuous stirring to remove the ethanol, yielding a final viscous ink of about 17% w/w solid in ethylene glycol, as seen in Fig. 5(a).

### Fabrication of the absorber sample

To make patterns on silicone, a stencil was fabricated with industrial laser cutting technology (JialiChuang Tech. LTD, Shenzhen, China). As seen in Fig. 5(b), the stencil was patterned according to the designed unit array as in Fig. 1(a). The to-be-printed parts were left as openings (dark parts in Fig. 1(a)). In absorber fabrication, shown in Fig. 5(c), the stencil was placed on silicone sheet. Both stencil and silicone sheet were fixed on a vacuum absorption surface. The ready-made graphene nano-flakes ink was put into a commercially available air blaster (Clasohlson Air Tool Kit 30–9875) and sputtered on silicone through stencil openings. After the sputtering process was finished, the stencil was removed and the as-designed printed patterns were left on the silicone sheet. The printed sample was then dried in oven for 15 min at 130 °C. The Raman and XRD (X-ray diffraction) spectrum analysis of the printed sample is given in Figures S2 and S3, respectively.

### Data availability

All data generated or analysed during this study are included in this published article and its supplementary information file.

## Additional Information

**How to cite this article**: Huang, X. *et al*. Experimental Demonstration of Printed Graphene Nano-flakes Enabled Flexible and Conformable Wideband Radar Absorbers. *Sci. Rep.* **6**, 38197; doi: 10.1038/srep38197 (2016).

**Publisher's note:** Springer Nature remains neutral with regard to jurisdictional claims in published maps and institutional affiliations.

## Supplementary Material

Supplementary Information
