# Supplementary material for: Experimental Demonstration of Printed Graphene Nano-flakes Enabled Flexible and Conformable Wideband Radar Absorbers
Source: Sci Rep. 2016 Dec 7;6:38197. doi: 10.1038/srep38197 (PMC5141498; doi:10.1038/srep38197)
Supplement: Supplementary Information [file srep38197-s1.pdf]

Experimental Demonstration of Printed Graphene Nano-flakes Enabled Flexible and Conformable Wideband Radar Absorbers (Supplementary Information)

Xianjun Huang, Kewen Pan, Zhirun Hu,[a)]

School of Electrical and Electronic Engineering, University of Manchester, Manchester, M13 9PL, UK

[(a)]Correspondence: Zhirun Hu (z.hu@manchester.ac.uk)

The cross sectional SEM view of the printed absorber is shown in Figure.S1. Clearly there are three layers from top to down, and they are protective layer, graphene nano-flake layer and silicone sheet in sequence. From the SEM, the printed graphene nano-flake layer has thickness of about 11μm. With the measured sheet resistance of this area Rs=21Ω, the conductivity of the layer can be calculated to be σ=4.33×10^3 S/m, from σ=1/(Rs×t). The protective layer (acrylic protective lacquer) was sputtered on for resistive to rub, dust, water, etc.

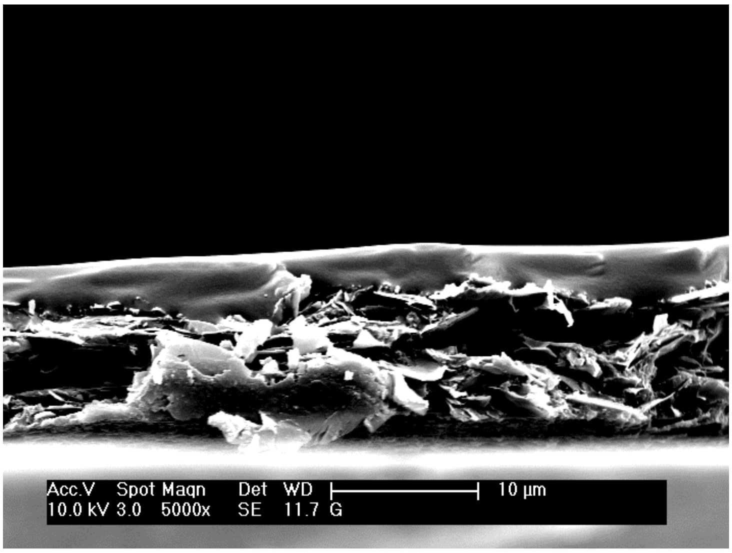

Figure.S1. The cross-sectional view of the graphene nano-flakes ink printed radar absorber.

The Raman and XRD spectrum analysis are also carried out as shown in Figure.S2 and Figure.S3 to characterize the printed sample.

In Figure.S2, chemical characterization of the printed graphene nano-flakes layer is done by Raman spectroscopy. This is a typical Raman characterization of graphene nano-flakes ink printed thin layer [1-3], and low quantity of defects is confirmed with this Raman characterization.

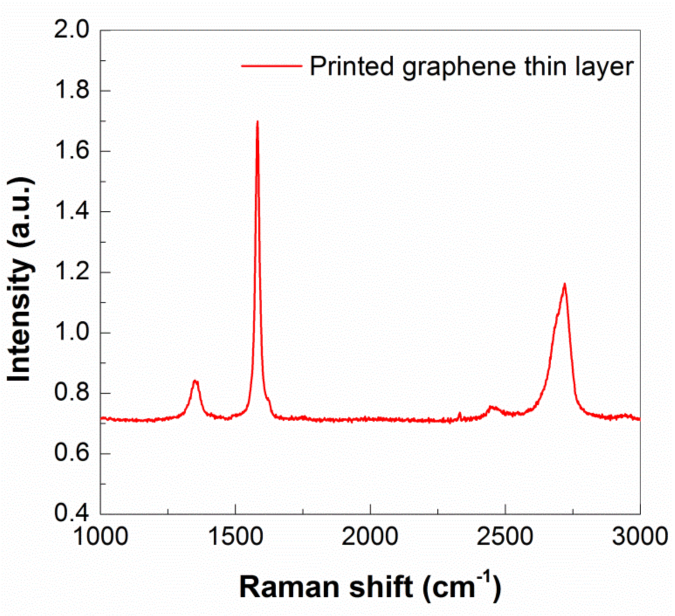

Figure.S2 Raman Spectrum of the graphene nano-flakes ink printed sample.

Besides, the XRD spectrum of the screen printed thin layer with graphene nano-flakes ink is shown in Figure.S3. From the XRD spectra, only graphite crystalline peak was observed. The peak at 2Θ = 26.7° corresponds to the 002 plane of graphite layers with an inter-layer spacing of 0.334 nm [2].

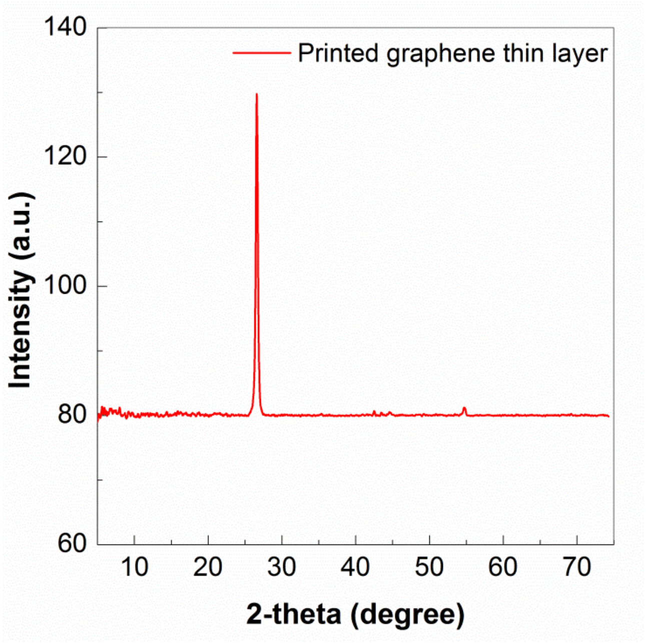

Figure.S3 XRD spectrum of the graphene nano-flakes ink printed sample.

Reference

[1] https://graphene-supermarket.com/Conductive-Graphene-Dispersion-200.html.

[2] Del, Sepideh Khandan, et al. "Optimizing the optical and electrical properties of graphene ink thin films by laser-annealing." 2D Materials 2.1 (2015): 011003..

[3] Arapov, Kirill, et al. "Conductivity Enhancement of Binder-Based Graphene Inks by Photonic Annealing and Subsequent Compression Rolling."Advanced Engineering Materials (2016).
